# A Benefit/Risk Assessment of Intrathecal Ziconotide in Chronic Pain: A Narrative Review

**DOI:** 10.3390/jcm13061644

**Published:** 2024-03-13

**Authors:** Emanuele Rubiu, Francesco Restelli, Vittoria Nazzi, Elio Mazzapicchi, Giulio Bonomo, Pierlorenzo Veiceschi, Tommaso Alfiero, Gianluca Agresta, Davide Locatelli, Alessandro Dario

**Affiliations:** 1 Department of Neurosurgery, Fondazione IRCCS Istituto Neurologico Carlo Besta, 20133 Milan, Italy; emanuele.rubiu@unimi.it (E.R.); vittoria.nazzi@istituto-besta.it (V.N.); elio.mazzapicchi@unimi.it (E.M.); dott.giuliobonomo@gmail.com (G.B.); 2 Department of Neurosurgery, Ospedale di Circolo e Fondazione Macchi, 21100 Varese, Italy; pierloveiceschi@gmail.com (P.V.); gianluca.agresta9@gmail.com (G.A.); davide.locatelli@asst-settelaghi.it (D.L.); alessandro.dario@asst-settelaghi.it (A.D.); 3 Department of Neurosurgery, Legnano Hospital, 20025 Legnano, Italy; alfiero.tommaso@gmail.com

**Keywords:** Ziconotide, chronic pain, intrathecal analgesia

## Abstract

Background: Ziconotide is an intrathecal drug administered for the treatment of chronic pain. The current literature lacks an exhaustive benefit/risk assessment on this drug. We herein focus on Ziconotide’s pharmacology and clinical applications. Methods: Literature research was conducted to identify studies on Ziconotide administration for the treatment of chronic pain, published between January 1990 and March 2023 and located via PubMed, Embase, Medline, Cinahl, and Web of Science, using the following keywords: Ziconotide, Omega conotoxin, Prialt, SNX-111, intrathecal therapy, and neuropathic pain. Only publications written in English were selected. Results: Among the 86 selected studies, we found 4 Randomized Controlled Trials (RCTs) and 3 prospective long-term studies concerning the intrathecal use of Ziconotide as a monotherapy in chronic pain. Other studies described the intrathecal infusion of Ziconotide combined with other drugs. Overall, Ziconotide has been proved to have strong efficacy for relieving chronic pain, although patients with co-morbid psychiatric disorders require a careful monitoring when treated with Ziconotide. Conclusions: Overall, the use of Ziconotide, as a monotherapy or in conjunction with other therapies for the treatment of chronic pain, was reported to be efficacious. Overall, its use in patients with chronic pain refractory to other pharmacologic agents outweighs the possible adverse consequences, thus resulting in a favorable benefit/risk assessment.

## 1. Introduction

Non-malignant chronic pain affects approximately 50 million adults, with a 20% prevalence, in the United States (US), concurring with both rising healthcare costs and a loss of productivity [1]. This medical condition is responsible for a reduction in daily activities, dependence on opioids, and depression with an increased risk for suicide [2]. It was determined that 12%, 14%, 18%, 18%, and 31.5% of people evaluated in Australia [3], Scotland [4], South Africa [5,6], the Netherlands [6], and China [7], respectively, are affected by chronic pain.

The International Association for the Study of Pain (IASP) defines chronic pain as a condition lasting longer than three months [8]. Depending on its cause, chronic pain can be classified as either inflammatory or neuropathic. While inflammatory pain results from tissue injury, neuropathic pain is caused by damage to the somatosensory system, resulting in abnormal activation of nociceptive receptors, even in the absence of any stimuli [9]. Recent genetic, molecular, and cellular techniques have led to considerable progress in the understanding of pain pathophysiological mechanisms. The identification of ionic channels on nociceptive neurons [10] and the application of molecular genetics in the study of pain mechanisms have contributed to the understanding of molecular mechanisms involved in pain pathways; this, in turn, has fostered the identification of new targets for analgesic drug development [11].

The first therapeutic step in nociceptive chronic pain management is represented by the oral administration of conventional analgesics [12,13]. Such treatment options include non-steroidal anti-inflammatory drugs (NSAIDs) as first-line agents, followed by opioids. Considering neuropathic pain, first-line drugs include tricyclic antidepressants and calcium current-blocking gabapentinoids [12]. Opioids are commonly used as the second- or third-line therapies; being related to significant side effects such as constipation, nausea, and somnolence, as well as the development of tolerance and opioid-induced hyperalgesia. Additionally, long-term benefits are still unclear: only a quarter of patients experience a significant pain reduction after a 12-month period of therapy [14,15]. Besides oral pharmacotherapy, pain relief may be achieved via intrathecal drug delivery (IDD), which delivers drugs in the spinal intrathecal compartment through an intrathecal catheter connected to a subcutaneous pump. The main indications of such treatment in non-malignant chronic pain are pain-relief failure or intolerable side effects from systemic drugs [16].

Among other compounds, Ziconotide is the most recently discovered drug among the antihyperalgesic drugs administered via implantable pumps. It blocks the N-type voltage-sensitive calcium channels and prevents neurotransmitter release from primary nociceptive afferents, terminating in the superficial layers of the spinal cord (i.e., the Rexed laminae II and I).

The main objective of this narrative review is to carry on a comprehensive review on Ziconotide use in chronic pain to clearly evaluate its specific benefit/risk ratio.

## 2. Material and Methods

Literature review was conducted to identify studies on intrathecal Ziconotide administration and to provide a benefit/risk assessment for this drug. The collected articles were published between January 1990 and March 2023 on PubMed, Embase, Medline, Cinahl and Web of Science. The following keywords were searched: “Ziconotide”, “Omega conotoxin”, “Prialt”, “SNX-111”, “Intrathecal therapy”. All papers were screened independently to identify their abstract, title relevance and contents by three authors (PV, GA and FR). In the case of discrepancy during the selection process, a consultation with the senior author (AD) was conducted. After selection of the full texts, study characteristics were extracted. The information collected included authors, publication year, country, study design, patients’ demographic information (age, sex, races, and ethnicities), type of pain, dose of Ziconotide, drug titration, and results. We voluntarily included both controlled (randomized or non-randomized) and uncontrolled studies (case series or case reports) in this review. Specific exclusion criteria were papers that were not written in English, papers dealing with Ziconotide use in acute or subacute pain, papers with massive loss at follow-up, and incomplete results.

## 3. Results and Discussion

**Figure 1:**
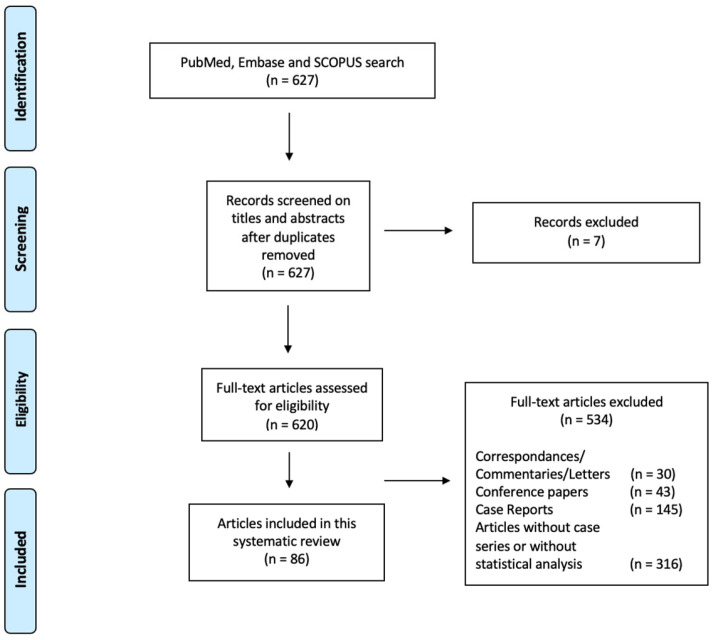
The flowchart of search hits with selection phases, from the initial search and the follow-up search, resulting in the total amount of 86 included articles.

Overall, we were able to find 627 works that, after removal of duplicates and removal of papers on the basis of the abovementioned exclusion criteria, resulted in the final analysis of 86 papers (Figure 1). Specifically, we reported four Randomized Controlled Trials (RCTs) and three prospective long-term studies, whose results are included in Table 1 (Ziconotide as a monotherapy), while various other studies described the intrathecal infusion of Ziconotide combined with other drugs. The side effects are reported in Table 2.

### 3.1. Ziconotide as a Monotherapy Regimen

We found four RCTs in which Ziconotide was used as monotherapy [17,18,19,27]. The first study to check for the efficacy and safety of intrathecal (IT) Ziconotide monotherapy was a randomized, placebo-controlled trial regarding patients with cancer (the tumor types were breast (11), lung (13), colorectal (10), prostate (5), myelogenous and lymphatic (7), and skin (3), tumors) or AIDS-related chronic pain [18]. In this study, despite its brief duration, titration, and maintenance phases (5 to 6 days and 5 days for responders, respectively), Ziconotide was demonstrated to reduce the mean visual analogue scale of pain intensity (VASPI) scores, improving pain intensity by 53% in drug-treated patients compared to the placebo group (*p* < 0.001) [18]. Ziconotide efficacy was also confirmed during the following maintenance phase [18]. Moreover, in patients randomized to placebo that crossed over to the treatment group, the mean reduction in the VASPI score was 45% [18]. To note, the titration dosages and titrations schedule used in this study requested a change to a tolerable dosing regimen (Table 1). The most common reported adverse events (AEs) included dizziness, postural hypotension, and nystagmus [18].

To evaluate the eventual tolerability of a slower Ziconotide titration schedule, in 2006, Rauck and colleagues presented the results of a randomized, placebo-controlled trial in patients affected by non-cancer-related chronic pain [17]. In this trial, a slower titration schedule was used (starting dose of 0.1 μg per hour with upward titration by 0.05 to 0.1 μg per hour; no more than once time every 24 h) [17]. In addition, the maximum dose of Ziconotide was much lower than in the previous study (0.8 μg per hour versus 2.4 μg per hour) [17]. During the three-week treatment period the VASPI scores improved in the treatment group by 12% compared with the placebo group (*p* < 0.05) [17]. To note, during the treatment period, 92.9% of patients receiving Ziconotide and 82.4% of patients in the placebo group reported mild to moderate AEs, such as dizziness, confusion, ataxia, abnormal gait, and memory impairment [17]. In 2016, Bruel and Burton published a post hoc pooled analysis of the aforementioned randomized trials, concentrating on patients with cancer-related pain, confirming that mean percentage improvement in VASPI score was significantly greater in Ziconotide group compared with the placebo group [28].

In a 2006 RCT by Wallace and colleagues, among a cohort of 169 Ziconotide-treated patients affected by severe non-malignant pain, a treatment of a 6-day period with starting and maximum doses of 0.1 μg per hour and 2.4 μg per hour, respectively, allowed a mean percent reduction in the VASPI score from the baseline of 31.2% in Ziconotide patients and 6.0% in placebo patients (*p* < 0.001) [19].

Additional evidence on the beneficial role of Ziconotide in the treatment of neuropathic pain comes from the clinical trial by Shao and colleagues, who showed that IT Ziconotide is able to improve pain and also emotional well-being [29]. The authors included 14 patients, who were followed for a mean period of time of 10.91 +/− 0.7 months, in which an improvement not only in the nociceptive symptomatology but also in pain catastrophizing, disability, and emotional well-being [29]. Despite its importance, this study and all literature is still lacking long-term efficacy and safety assessments for Ziconotide [30].

Three large prospective, long-term studies were previously published using IT Ziconotide. Webster et al. [25] investigated IT Ziconotide long-term administration in an open-label, multicenter study, demonstrating its safety and sustained efficacy for up to three years in 78 malignant and non-malignant chronic pain patients, with a mean dose of 6.48 μg per day, which is similar to the mean dose of 6.9 μg per day previously reported [17]. VASPI scores remained stable during the study, suggesting no evidence of increased pain with increased duration of drug exposure; long-term Ziconotide treatment appeared to be well tolerated as well. In another prospective study conducted on 155 patients, with 48 cancer patients (tumor types were not available) and 107 non-cancer patients, with chronic pain, AEs were reported in 147 out of 155 patients (94.8%) but the VASPI scores decreased by a mean of 36.9% from the baseline in the short term [20]. Moreover, in a study involving 644 patients, with only 16 of them suffering from cancer-related chronic pain (tumor types not available) just 119 patients received IT Ziconotide for over a year but, unfortunately, VASPI scores were recorded just from the baseline to the two-month checkpoint [21]. Hence, the above-described clinical studies have suggested that Ziconotide monotherapy may be efficacious in malignant and non-malignant long term pain management [24].

In a report from the Italian registry of Ziconotide, a long-term retrospective observational database involving cancer and non-cancer patients, a total of 104 patients were considered: 55 patients, with 20 suffering from chronic pain related to cancer, received intrathecal Ziconotide; and 49 patients, with 12 of them affected by cancer, received placebo. Regarding the tumor type, the authors included 4 patients affected by breast cancer, 8 patients affected by lung cancer, 4 patients affected by colorectal cancer, and 10 other patients affected by unspecified types of cancers. The study disclosed a good efficacy of Ziconotide in malignant and non-malignant intractable pain [22]. Pain intensity was tested after one month of treatment and 69.23% of patients reported a pain intensity reduction of at least 30%, with a mean dose of 4.36 μg per day and a treatment period of 53 days [22]. Furthermore, 53.84% experienced a 50% pain intensity reduction and almost all patients experienced such reduction within a mean treatment period of 82 days [22]. Cancer patients attained 20–50% pain reduction within one month of treatment on average, while non-cancer patients obtained a 20–50% pain reduction within 3 months [22]. The main reasons for treatment interruptions were adverse events (18.26% of cases) and uncontrolled pain (6.73% of cases). Most patients with cancer-related pain reported a decrease of at least 50% in pain intensity with a reported mean Ziconotide dose of 5.5 μg per day [22].

According to the above-reported results and to the Polyanalgesic Consensus Conference (PACC) guidelines [31], it is now recommended to initiate IT Ziconotide at no more than 2.4 μg per day, with upward titration by up to 2.4 μg per hour., and at intervals of no more than two to three times per week. Two case reports have suggested a rapid dose escalation in patients with intractable cancer-related neuropathic pain without serious side effects; such patients were affected by severe intractable back pain from spinal anaplastic ependymoma and by neuropathic pain from metastatic brain cancer, respectively [32,33].

Regarding trials of various methods, different strategies are extensively described in the 2012 PACC guidelines [34]. In the last update of such an expert panel, light was shed on a new concept of flex-dosing trialing. New bolus flexing dosages were proposed to potentially improve the tolerability and efficacy of Ziconotide, according to Pope and Deer back in 2015 [35]. In their study, after trialing, non-cancer patients with intractable pain were treated with a flex-dosing strategy, weighted during nocturnal dosing. The numerical rating scale decreased from a mean of 9.06 to 1.8, while all the patients met the endpoint of the study in terms of tolerability at three months [35]. Bolus injections of Ziconotide were then proposed and applied in clinical practice for non-cancer patients [36,37,38]. For example, in a study by Mohammad and colleagues, similarly to studies by Rosenblum and Grisby, bolus administration of Ziconotide appeared to be safe and effective in predicting the response to Ziconotide infusion [36]. On the contrary, Backryd et al. found that even though Ziconotide bolus injections are feasible, the proportion of responders is low, showing that the predictive power of Ziconotide bolus trialing methods remains unclear as a protocol [37]. It is far from the intentions of this paper to discuss the trialing indications, methods, and results, but, while it is generally agreed that a trial should be performed prior to implantation, there is no actual consensus about the type of trial that should be used. Bolus administration requires a shorter duration of hospitalization and a lower cost, whereas the main disadvantage may be the high placebo response; moreover, infusion may better mimic the slow titration effects of a chronic drug infusion [34] and the pharmacological profile of Ziconotide (slow tissue penetration due to high hydrophilicity) calls the rationale for bolus trialing into question.

### 3.2. Ziconotide in a Polytherapy Regimen

Other standard-of-care IT agents, such as the local anesthetic bupivacaine, alpha-2 agonists, and the opioids hydromorphone and fentanyl, are often used off-label [39], sometimes in combination with morphine or Ziconotide. Furthermore, Ziconotide infusion strategies have been suggested to improve the monotherapy longevity [39]. Looking at drug combinations, in recent years, many studies have suggested that they have a positive effect on pain relief.

IT infusions of Ziconotide and morphine were tested in an open-label, multicentric study by Webster and colleagues, and proved to reduce pain in patients with suboptimal pain relief on Ziconotide monotherapy. In addition, a decrease in systemic opioid consumption was demonstrated [40]. In a prospective observational study by Alicino and colleagues [41] conducted on 20 refractory pain patients with bone metastasis, Ziconotide was started at a dose of 2.4 μg per day, followed by increases of 1.2 μg per day at intervals of at least 7 days. Concurrently, an initial IT morphine daily dose was calculated based on its oral daily dose. A significant reduction in VASPI score was seen as early as 2 days (*p* < 0.001) after treatment administration. A significant change in VASPI score was also seen at 7 days and 28 days (*p* < 0.001 in both situations). Four patients experienced mild adverse events related to the studied drugs [41].

In a study by de la Calle Gil [42], an IT combination of Ziconotide and morphine was used in cancer patients with neuropathic pain, suggesting that adding Ziconotide to IT morphine alone may increase pain relief in such patients [42].

In an observational study by Dupoiron et al. [26], 77 patients with chronic intractable cancer-related pain were treated with an aggressive Ziconotide dose titration (starting at 1.0 μg per day, titrated by 0.25 to 0.5 μg per day every 2 days) in combination with other IT agents, such as morphine, ropivacaine, and clonidine. Such combinations reduced pain, as proved by the reduction in the NRS score from 8.1 at baseline to 4.1 in one month [26].

Moreover, Deer and colleagues demonstrated a good efficacy in pain treatment with IT combination therapies [43]. Nevertheless, in 2014, Hayek et al. [44] conducted a 24 month follow-up study on chronic non-cancer patients where Ziconotide was added to the IT infusion mixture, and a high incidence of AEs was seen, shedding light on the need to have more prospective and randomized trials on multi-drug regimens including Ziconotide.

Looking at the combination of Ziconotide and hydromorphone, one case report documented the good pain control in a 15-month follow-up of a 23-year-old woman with chronic pain caused by a traumatic spinal cord injury [45]. Ziconotide and clonidine, a lipophilic alfa2-adrenergic agonist, have been mainly tested in preclinical models [46,47]. Even for bupivacaine, a local anesthetic, the results of IT combined therapies have been studied in preclinical models [46]. Looking at the addition of Baclofen to Ziconotide monotherapy, some studies suggest that such association may help to manage spasticity-related pain [48,49]. A multi-combination therapy in pediatric patients was reported in a case report for pediatric population [50].

In recent years, Ziconotide has also been intraventricularly administered effectively as an “off-label drug” [51]. Ziconotide was used to treat complex regional pain syndromes in seven patients [52] with inadequate pain relief after multiple conventional therapies, suggesting a promising efficacy in patients affected by complex conditions [50] and in chronic migraine headaches [53]. In 2016, Voirin [54] reported a case in which IT Ziconotide was used as a second-line treatment after failure of motor cortex stimulation in a neuropathic chronic pain syndrome. Russo and colleagues [55] used IT Ziconotide in a case of primary erythromelalgia, resistant to conventional therapies, obtaining a dramatic clinical improvement after one week from the drug administration. Trigeminal neuralgia actually proved to be responsive to Ziconotide, but further studies are definitely needed to better define off-label indications of such IT drugs [56].

### 3.3. Risk Evaluation

The most common Ziconotide-related adverse events (AEs) include dizziness, nausea, nystagmus, somnolence, urinary retention, amblyopia, hypotension, confusion, asthenia, and abnormal gait. A complete list of the reported adverse effects is reported in Table 2. Many of them were described after initial drug bolus, while most of them occurred during the first two weeks of treatment [17,21]. AEs can be divided into cognitive–neuropsychiatric and systemic reactions. All effects are dose-related and could be minimized starting from low doses and gradually titrating upward. In fact, following the 2007 PACC [57], Ziconotide has been recommended as a first-line agent in the algorithm for nociceptive, mixed and neuropathic pain, starting at a lower dosage of 0.5 μg per day with 0.5 μg increments every week for titration. The maximum recommended dose is 19.2 μg per day (0.8 μg per hour) [57].

#### 3.3.1. Cognitive and Neuropsychiatric Adverse Reaction

Psychiatric symptoms include hallucinations (12%), paranoid reactions (3%), hostility (2%), delirium (2%), psychosis (1%), and manic reactions (0.4%) [3]. Ziconotide may also cause or worsen depression. Several case reports found a higher incidence of suicide, suicide attempts, and suicide ideations in Prialt-treated patients, and all symptoms remitted immediately after drug discontinuation [58]. These cases substantiate the suspicion of a causal relationship between Ziconotide and suicidality even in symptom-free patients with a history of depression. Other reports also sustain these findings [59,60]. Therefore, a comprehensive psychiatric evaluation is recommended before starting and during Ziconotide treatment [61].

Use of Prialt has also been associated with cognitive impairment and a decreased state of alert and responsiveness [7,8]. The most common reported cognitive AEs include confusion (33%), memory impairment (22%), speech disorder (14%), aphasia (12%), thinking abnormal (8%), and amnesia (1%). Cognitive impairment usually appears gradually after starting the treatment. Elderly patients but also patients undergoing concomitant antiepileptic, neuroleptic, sedative, or diuretic treatments may be at higher risk of depressed levels of consciousness [10]. Ziconotide administration should be suspended in patients with risk of reduced consciousness. Other nervous system disorders include areflexia, gait impaired, burning sensation, coordination abnormalities, disturbance in attention, dizziness, dysarthria, hypoesthesia, mental impairment, and sedation [17,21].

#### 3.3.2. Systemic AEs

AEs have been reported in 2% or greater of patients participating in the above-cited clinical studies [17,21]. Such events are represented by falls, fatigue, lethargy, special senses disturbances, such as diplopia and blurred vision; urogenital alterations, such as urinary retention and dysuria; and digestive symptoms, such as abdominal pain, constipation, diarrhea, nausea, or vomiting [17,21]. Bradypnea, decreased oxygen saturation, syncope, hypotension, muscle cramp, weakness, and myalgia have also been reported [17,21].

Patients taking Prialt^®^ may suffer elevations in serum creatine kinase (CK) as well. In fact, in several clinical studies, a shift from normal at baseline to above normal was reported. Rauck et al. demonstrated significant elevation of CK serum values up to three times the upper limit of 198 IU/L in 5 out of 112 patients receiving IT Ziconotide. One patient also experienced hypokalemia, but the authors did not relate the alternation to IT Ziconotide [21].

Wallace et al. reported a case of acute tubular necrosis as a result of Ziconotide-induced rhabdomyolysis and myoglobinuria with a peak CK level of more than 16,000 IU/L. This patient, however, had a history of opioid abuse and prolonged immobility, so the rhabdomyolysis was attributed to muscle compression during unresponsive states [21].

Horazeck and colleagues [62] reported a case of acute rhabdomyolysis in a 71-year-old woman with long-term exposure to IT Ziconotide after IT bolus injection of Ziconotide. The patient suffered from failed back surgery syndrome (FBSS)-related chronic neuropathic pain and received IT Ziconotide for 2 years. When the patients developed neurological side effects, the pump medication was shifted to morphine, which failed to provide adequate analgesia even with dose titration. A single IT bolus of Ziconotide was then administered and resulted in excellent pain relief. Two months later, the patient received a second Ziconotide injection and 16 h, following which she was admitted to the local emergency department complaining of nausea, vomiting, diarrhea, and myalgia. Blood tests disclosed significantly increased CK levels (3594 IU/L) without signs of myoglobinuria, suggesting mild rhabdomyolysis. The patient received intravenous hydration and close observation since the normalization of the CK [62].

All these reports suggest that patients undergoing treatment with Ziconotide should be periodically clinically and biochemically monitored, looking for symptoms like myalgias, myasthenia, muscle cramps, asthenia, and dosing serum CK. If these symptoms persist and/or CK levels become elevated, reducing or discontinue Prialt^®^ administration is mandatory. One case of severe cardio-vascular toxicity was recorded by Heifets. Following two Ziconotide infusions, a 42-year-old woman developed a severe headache, intermittent emesis, difficult-to-clear airway, a hemodynamically tolerated tachycardia–bradycardia syndrome, and uncompensated respiratory acidosis. The event was explained as an acute Ziconotide side-effect and required support therapy [63].

#### 3.3.3. Overdose Risk

A catastrophic iatrogenic complication may occur during the management of the device, e.g., during the programming of the drug dose and concentration or during the injection into the pump reservoir. IT administration requires a lumbar puncture to evacuate CSF and exchange it with artificial fluid: in fact, there is no known antidote to Ziconotide [34]. General medical supportive measures should be administered to patients who receive an overdose until the exaggerated pharmacological effects of the drug have resolved. Treatment for an overdose is hospitalization, when needed, and symptom-related supportive care.

Pozzi et al. described dyskinesia in a 15-year-old male suffering spastic dystonic tetraparesis after Ziconotide and Baclofen combination therapy. After 7 years of successful treatment with high-dose IT baclofen, the patient’s chronic pain became insensitive to analgesics. Hence, Ziconotide was added to the infusion reservoir, with a total volume of 20 mL for a final concentration of 1.9 mg/mL for baclofen and 1 μg/mL Ziconotide. After 2 days, the patient displayed involuntary dyskinetic movements, which affected the head and upper limbs. Both the onset and the cessation of the ADR happened within a period of 2 days and the symptoms were considered specifically related to Ziconotide. The authors concluded that baclofen activates the GABA-B receptor that indirectly regulates the opening of N-type voltage-sensitive calcium channels (VSCCs), which are also targeted by Ziconotide: hence, the two drugs may synergize, allowing Ziconotide to exert a toxic effect [64].

### 3.4. Comparison with Alternative Therapies

The FDA-approved intratechal medications for pain control are Morphine and Ziconotide. Both have demonstrated efficacy in alleviating pain in patients with cancer-related symptoms, although, for both, only few randomized, controlled studies are available [31]. One particular advantage of Ziconotide is represented by the fact that it does not interact with the opioid receptor [65]. As a result, no endocrine side effects, which are common with morphine administration, are seen. Such adverse effects include a loss of libido, falling testosterone levels in men, and risk of spinal osteoporosis [66], combined with hypogonadotropic hypogonadism and amenorrhea or irregular menstrual cycles [67,68]. Vitale and colleagues [69] hold that Ziconotide is the only drug of choice for younger patients. Contrarily, patients who have been receiving IT morphine therapy have a high incidence of hyperalgesia [20] and tolerance does not occur [20,24,70]. In a case study of an opioid refractory patient who switched to IT Ziconotide, no signs of pharmacological tolerance, neurotoxicity, or cardiovascular side effects were discovered [24]. Hence, it is important to realize how the lack of addiction, lack of withdrawal effects, opioid-induced hyperalgesia, and other systemic effects common with morphine are absent with Ziconotide [69]. These factors have firmly placed it in the first line for the Polyanalgesic guidelines in IT drugs [66].

Although morphine and Ziconotide are currently the only FDA-approved pain medications for IT use, a variety of monotherapies or combination agents, including hydromorphone, fentanyl, sufentanil, bupivacaine, clonidine, and baclofen, are in use [39].

### 3.5. Benefit–Risk Evaluation

Despite the encouraging studies on the administration of Ziconotide by the non-IT route [71,72] currently the only administration is that by chronic IT infusion.

This method of administration is obviously linked to surgical implantation of the pump, which is not risk-free. However, among the drugs used intrathecally, Ziconotide is the only one that did not cause the formation of IT granulomas [73,74]. The data of the PRIZM registry, in which a total of 93 patients were enrolled, with 4 of them affected by malignant chronic pain, with no specification about the type of tumor, suggest effectiveness when Ziconotide is infused as the first agent [23].

The most important side effects seem to be psychiatric disorders that can worsen when already present [61] and the elevation of CK serum values, which can lead to consequences from a metabolic viewpoint [62]. Since the adverse consequences of IT Ziconotide infusion do not exceed the benefits in patients with chronic drug-resistant pain compared to oral or intramuscular–venous administration, the benefit/risk assessment is favorable.

### 3.6. Ziconotide: Mechanism of Action and Pharmacological Assessment

Ziconotide represents the most recently discovered drug among the antihyperalgesic drugs administered via implantable pumps. Its mechanism of action implies the blockage of N-type voltage-sensitive calcium channels, thus preventing neurotransmitter release from primary nociceptive afferents, which terminate in the superficial layers of the spinal cord (i.e., Rexed laminae II and I) [75]. Based on the pioneering work conducted by Olivera et al. in 1960 regarding the effects of marine snail toxins [76], Ziconotide was developed as an artificial drug in the late 1980s. This novel non-opioid analgesic is a synthetic version of v-conotoxin MVIIA (v-MVIIA), a peptide found in the venom of the Conus magus, a fish-eating marine snail [75]. The U.S. Food and Drug Administration (FDA) approved it under the name of “Prialt” in 2004 for the management of long-term neuropathic pain. In 2005, the drug was also approved by the European Commission. Due to its limited ability to cross the blood–brain barrier, Ziconotide must be administered intrathecally (IT). Elan Pharmaceuticals originally marketed the “Ziconotide Intrathecal Infusion” system as Prialt^®^, which implies a continuous delivery by means of a programmable surgically implanted infusion device. The use of an infusion pump allows to titrate the dose of drugs according to patients’ needs, achieving optimal balance between analgesic efficacy and side effects.

Regarding its clinical indications, in current clinical practice, IT Ziconotide represents the first-line treatment in chronic non-malignant and localized nociceptive or neuropathic pain. Concerning non-localized nociceptive or neuropathic pain, IT Ziconotide represents the second line of treatment [39]. Furthermore, Ziconotide is recommended as the first choice of treatment for patients taking more than 120 equivalent of morphine daily, with no history of psychosis [39]. In patients afflicted by neuropathic pain, hypersensitivity results from the upregulation of nociceptors’ voltage-gated calcium channels (VGCCs) by endogenous factors. Several analgesic drugs reduce pain by inhibiting such ionic channels [77]. Six subtypes of voltage-activated calcium-permeable ion channels have been identified throughout the nervous system, named as L, N, P/Q, R and T channels [78,79]. N-, P/Q- and R-type channels are located at synaptic sites and are involved in both excitatory and inhibitory neurotransmitter release. N-type and P/Q-type calcium channels are localized predominantly on pre-synaptic fibers [80], playing a critical role in the biochemical cascade of events that leads to the exocytotic release of neurotransmitters [25]. The N-type channels are also distributed throughout the dorsal horn, being the predominant subtype in I and II Rexed laminae [81].

Although the mechanism of action has not been established in humans yet, the results from animal models suggest that Ziconotide selectively and reversibly binds to N-type voltage-sensitive calcium channels. The drug may produce analgesia by blocking neuropeptides and glutamate release from nociceptive afferent nerves in the dorsal horn of the spinal cord [82]. More deeply, Ziconotide binds to N-type calcium channels located in the primary nociceptive afferent neurons of spinal cord Rexed laminae II and I [24]. By interfering with such neurotransmission, Ziconotide reduces pain signaling via the spinothalamic tracts [83]. Molecularly, the target is represented by the N-type calcium channel, also known as CaV2.2, a high-voltage-activated channel that contains the 1B subunit. Evidence suggests that, with the exception of the 2δ subunit, which binds gabapentin and pregabalin, the 1B subunit contains most of the pharmacologically relevant binding sites of this calcium channel.

The pharmacokinetics of IT Ziconotide have been explored in both animal and human clinical studies. In Beagle dogs, drug pharmacokinetics were monitored with a single IT bolus injection at the dose of 10 μg in 1 mL, followed by continuous IT infusions at the dose of 1 μg in the first hour, and then at 5 μg with a rate of 100 μL per hour; each for 48 h) [2]. After IT administration, the cerebrospinal fluid (CSF) volume of distribution for Ziconotide, being of around 99.2 mL, approximates the estimated total human CSF volume, i.e., 150 mL. Median CSF clearance (CL) of Ziconotide, being of 0.26 mL per minute, approximates the adult human CSF daily turnover rate, which is 500 mL. No adverse events were found following a single, 1 h IT infusion of Ziconotide, with vital signs remaining relatively unchanged. Based on its elimination half-life (4.5 h), Ziconotide should reach steady-state levels in spinal CSF during 24 h. The discrepancy between the time to steady-state concentration in the CSF and the time to the onset of effects suggests that the distribution of Ziconotide into the spinal and brain tissues significantly slows the distribution into the CSF [82]. During 5 or 6 days of continuous IT infusions, at infusion rates ranging from 0.1 to 7.0 ng per hour, plasmatic drug concentrations could not be quantified in 56% of patients using an assay with a lower limit of detection of approximately 0.04 ng per mL [82]. Predictably, patients requiring higher IT infusion dose rates were more likely to have quantifiable Ziconotide levels in their plasma [82]. Such plasmatic levels remained stable for up to 9 months after several months of IT infusion [82]. In the above-described study, the cumulative CSF exposure to Ziconotide, measured as the CSF area under the concentration–time curve, was significantly predictive of pain relief [82].

Ziconotide binds to human plasma proteins for about 50% of its concentration [82]. The mean CSF volume of distribution (Vd) of Ziconotide, following IT administration, approximates the estimated total CSF volume (140 mL) [20]. Ziconotide binds to plasma protein following its passage from the IT space to the bloodstream, where it is cleaved by various ubiquitous proteases expressed in kidney, liver, lung, and muscle tissues [82]. Moreover, it should be noted that in in vitro studies, for both human and animal CSFs, blood exhibits minimal hydrolytic activity toward Ziconotide [82].

Regarding its clearance (CL), the three possible CL routes of a drug from the CSF are represented by (a) the local uptake into the spinal cord, (b) the rostro-caudal bulk flow of CSF out of the Central Nervous System (CNS), (c) and the transdural penetration followed by absorption into blood [82,84]. Ziconotide, which has an uptake speed of approximately 0.26 mL/min, approximates the adult human CSF turnover rate, providing evidence that the primary mechanism for Ziconotide CL is bulk CSF flow, rather than a metabolic process. The terminal half-life of Ziconotide in CSF after IT administration is around 4.6 h (ranging from 2.9 to 6.5 h) [85]. In pharmacodynamic studies on patients affected by chronic pain, the analgesic effect of IT Ziconotide was dose-related and increased over time, suggesting the presence of a time lag in reaching the maximal response [82]. Based on its elimination half-life (4.5 h), Ziconotide should reach steady-state levels in the spinal CSF in 24 h [82]. The apparent discrepancy between the bolus IT administration of Ziconotide and its pharmacodynamic effects reflects the slow penetration of its large hydrophilic molecules of CNS parenchyma [82]. Following continuous administration, Ziconotide is not associated with dose tolerance. Moreover, tolerance to morphine is not related with cross-tolerance to Ziconotide [85]. Similarly, Ziconotide neither prevents morphine tolerance nor potentiates morphine-induced respiratory depression. Notwithstanding, Ziconotide has been shown to potentiate opioid-induced reduced peristalsis. As Ziconotide does not prevent or ameliorate opioid withdrawal symptoms, during IT opioid withdrawal, opioid dosages should be tapered and substituted with pharmacologically equivalent dosages of oral opioids [85]. In the case of opioid treatment failure, concomitant treatment with both oral opioid and IT drugs different from Ziconotide may not be an effective long-term strategy. However, IT Ziconotide appeared to hasten the decrease in oral opioid intake, whereas bupivacaine paradoxically increased the oral opioid intake [85].

### 3.7. Benefit Evaluation

From the data analysis, it was possible to evaluate two different types of treatments: Ziconotide monotherapy and Ziconotide administration in conjunction with one or more drugs.

## 4. Conclusions

From the analysis of the available literature, although the benefit/risk assessment of Ziconotide in chronic pain appears to be favorable when used as a monotherapy, specific long-term prospective randomized studies are still lacking. Due to the fact that many investigators reported high frequencies of treatment discontinuation, the real magnitude of its long-term effect in common clinical practice is difficult to determine. Moreover, the long-term evaluation of infusions with Ziconotide associated with other IT drugs is lacking both in terms of number and the quality of present studies. Future research should focus on the effective long-term use of Ziconotide in diverse chronic pain populations.

## Figures and Tables

**Table 1:** Intrathecal ziconotide monotherapy in randomized controlled trials and open-label studies. Legend: AEs: adverse events; h: hour/hours; MDA: maximum dose allowed; pts: patients; SD: starting dose; UT: upward titration; ZNT: Ziconotide).

Authors	Type of Study	Patients	Dosing/Titration	Relevant Results
Rauck et al. [17]	Short-term double-blind randomized, placebo-controlled trial	Any etiology of refractory pain patients (112 ZNT, 108 placebo)	SD: 0.1 mcg/h UT: 0-05-0.1 mch/h Increments at intervals more than 24 h to analgesic effect or AEs, possible downward titration to improve tolerability Maximum dose: 0.9 mch/h	Mean VASPI scores improved from baseline by 14.7% in ZNT group and by 7.2% in placebo group
Staats et al. [18]	Short-term double-blind randomized, placebo-controlled trial	Patients with refractory pain, with cancer or AIDS (71 ZNT, 40 placebo)	First 48 patients: SD: 5 ng/Kg/h then changed to 0.4 mcg/h UT: once every 12 h to max tolerated dose Subsequent 60 patients: SD < 0.1 mcg/h UT: once every 24 h to analgesic effect or AEs Maximum dose: 2.4 mcg/h	Mean VASPI scores improved by 53.1% in the ZNT group and by 18.1% in placebo group
Wallace et al. [19]	Short-term, double-blind, randomized, placebo-controlled trial	Non-cancer refractory pain patients (175 ZNT, 89 placebo)	First 65 patients SD: 0.4 mch/h UT: once every 24 h Maximum dose: 7 mcg/h Subsequent 199 patients: SD: 0.1 mch/h UT: once every 24 h to analgesic effect or AEs Maxiumum dose: 2.4 mcg/h	Mean VASPI scores improved by 31.2% in ZNT grioiup by 6% in placebo group (from baseline to end of titration period, day 6)
Ellis et al. [20]	Long-term, open-label study, continuation (RCT) of Staats and Wallace studies	Responders to ZNT in previous RCTs studies by Staats and Wallace (155 pts)	Pts maintained on their established dose for 30 days if analgesic effect present and AEs acceptable; upward/downward titration based on analgesic effect/AEs; maximum doses allowed: 2-fold increase per 12 h period	Mean VASPI scores improved from baseline by 31.8–45.8% for 1–12 months
Wallace et al. [21]	Long-term, retrospective, open label study	Severe chronic pain from different etiologies (cancer, AIDS) or of non-cancerous cause with a demonstrable neurological basis (644 pts)	-SD: ≤2.4 mcg/day -UT: ≤2.4 mg/day No maximum dose definition	Among ≥50 mm VASPI scores after 1 month of therapy 32.7% had ≥30% mean VASPI score improvement
Raffaeli et al. [22]	Long-term, retrospective study	Refractory chronic pain of both cancer/non-cancer etiologies (104 pts)	Not available SD or UT schedule; mean initial ZNT dose 1.41 (0.61) mcg/day	Relationship between efficacy and dose (pts with pain intensity reduction of ≥10 and 50%, mean daily doses were 3.50 and 4.98 mcg/daily, respectively)
Deer et al. [23]	Long-term, open label, retrospective study, interim analysis	51 pts received ZNT as the first agent (FIP+) in pump, 42 not as first agent (FIP−)	-SD: no more than 0.1 mcg/hour -UT: ≤2.4 mcg/day (0.1 mcg/hour) at a frequency of no more than 2 to 3 times per week based on analgesic response and AEs	Improvement from baseline (PGIC score), was reported in 69.2% of FIP+ and 35.7% of FIP− patients at month 6 and 85.7% of FIP+ and 71.4% of FIP− patients at month 12

**Table 2:** Summary of ziconotide-related adverse events as demonstrated by different clinical trials, conducted with only ziconotide (not in combination). Total numbers and (%) of patients affected among the entire cohort in analysis.

Author	Staats et al. 2004 [18]	Rauch et al. 2006 [17]	Ver Donck et al. 2008 [24]	Ellis et al. 2008 [20]	Wallace et al. 2008 [21]	Webster et al. 2009 [25]	Deer et al. 2018 [23]	Raffaeli et al. 2011 [22]	Dupoiron et al. 2012 [26]
Number of Patients	71	112	71	155	644	78	51	105	77
Any Adverse Effect	70 (97.2)	104 (92.9)	43 (60.06)	147 (94.8)	587 (91.1%)	37 (52.1)	41 (80.4)	66 (63.46)	44 (57)
General Sympthoms	33 (45.8)								
Back Pain					1 (0.2)				
Chest Pain					1 (0.2)				
Fever	18 (25)								
Headache	11 (15.3)	17 (15.2)	5 (7.0)	20 (12.9)	1 (0.2)			4 (3.84)	
Asthenia	5 (6.9)	25 (22.3)	6 (8.5)	22 (14.2)			3 (5.9)	23 (22.11)	
Intentional Injury					1 (0.2)				
Pruritus	3 (4.2)	9 (8.0)							
Malignant Hyperthermia					1 (0.2)				
Neck Rigidity					1 (0.2)				
Overdose					1 (0.2)				
Pain	2 (2.8)	12 (10.7)		10 (6.5)	1 (0.2)				
Chills				17 (11.0)					
Diaphoresis									6 (8)
Cardiovascular System	24 (33.3)								
Syncope					2 (0.3)				
Electrocardiogram Abnormal					1 (0.2)				
Hypotension/Postural Hypotension	17 (23.6)				1 (0.2)			4 (3.84)	9 (12)
Hypertension	6 (8.3)							2 (1.92)	
Digestive System	42 (58.3)				310 (48.1)				
Diarrhea	5 (6.9)	21 (18.8)		8 (5.2)	1 (0.2)		3 (5.9)		
Various Ge disorders	1 (1.4)							6 (5.76)	
Constipation	9 (12.5)								
Oral cavity disorders				12 (7.7)				8 (7.69)	
Nausea + Vomiting	9 (12.5)				1 (0.2)				
Nausea	21 (29.2)	46 (41.1)	10 (14.1)	22 (14.2)	202 (31.4)		10 (19.6)	10 (9.61)	23 (30)
Vomiting	13 (18.1)	17 (15.2)	2 (2.8)						
Anorexia	5 (6.9)			13 (8.4)					
Metabolic/Nutritional Disorders	2 (2.8)				105 (16.3)				
Dehydration					2 (0.3)				
Peripheral Edema	1 (1.4)				1 (0.2)		7 (13.7)		
CK elevation		12 (10.7)		12 (7.7)	61 (9.5)			5 (4.8)	2 (3)
Musculoskeletal System	7 (9.7)								
Myositis					1 (0.2)				
Myasthenia	6 (8.3)		1 (1.4)	26 (16.8)					
Nervous System	60 (83.3)				512 (79.5)				
Confusion	15 (20.8)	20 (17.9)	3 (4.2)	67 (43.2)	183 (28.4)		5 (9.8)		12 (26)
Dizziness	36 (50)	53 (47.3)	22 (31)	50 (32.3)	275 (42.7)	6 (8.5)	7 (13.7)		
Mental Slowing					65 (10.1)				
Agitation				10 (6.5)	1 (0.2)				
Depression				12 (7.7)	1 (0.2)				
Anxiety				12 (7.7)					
Altered Mood								5 (4.8)	19 (24)
Abnormal Thinking	4 (5.6)				1 (0.2)				
Memory Impairment/Amnesia		13 (11.6)		39 (25.2)	158 (24.5)	8 (11.3)	5 (9.8)		
Other psychiatric disorders					2 (0.3)			6 (5.76)	
Delirium					5 (0.8)				
Hallucinations				16 (10.3)	87 (13.5)		5 (9.8)	8 (7.69)	
Somnolence	17 (23.6)	25 (22.3)	3 (4.2)	21 (13.5)	2 (0.3)	5 (7.0)	2 (3.9)		
Insomnia/sleepness	4 (5.6)	7 (6.3)						5 (4.8)	
Hostility/aggressivness	7 (9.7)			15 (9.7)	1 (0.2)	5 (7.0)		5 (4.8)	
Stupor					6 (0.9)				
Ataxia	4 (5.6)	18 (16.1)	3 (4.2)	20 (12.9)	58 (9.0)			13 (12.5)	
Abnormal Gait/Balance Disorders	9 (12.5)	17 (15.2)		36 (23.2)	96 (14.9)	4 (5.6)	4 (7.8)	21 (20.19)	
Altered muscle tone				8 (5.2)				15 (14.42)	
Tremor/Psychomotor disorders				15 (9.7)	1 (0.2)			36 (34.61)	
Nystagmus/Vertigo	33 (45.8)		5 (7.0)	42 (27.1)	138 (21.4)	6 (8.5)			7 (9)
Tinnitus			2 (2.8)					2 (1.92)	
Impaired verbal expression				26 (16.8)	56 (8.7)				6 (8)
Dysarthria					1 (0.2)				
Speech disorders				20 (12.9)	75 (11.6)	6 (8.5)			
Neurovegetative disorders								10 (9.61)	
Dysesthesia				8 (5.2)	1 (0.2)				
Hyporeflexia				13 (8.4)					
Sensory impairments				14 (9.0)				16 (15.38)	
Meningitis					1 (0.2)				
Respiratory System	14 (19.4)								
Dyspnea	4 (5.6)				1 (0.2)				
Hypoventilation/Hypoxia	1 (1.4)				1 (0.2)				
Lung Disorder	4 (5.6)				1 (0.2)				
Pneumonia	1 (1.4)								
Special Senses	9 (12.5)				201 (31.2)				
Blurred Vision			3 (4.2)	11 (7.1)	82 (12.7)				
Taste Perversion				17 (11.0)	54 (8.4)				
Abnormal Vision	4 (5.6)			10 (6.5)					7 (9)
Urogenital System	23 (31.9)				134 (20.8)				
Urinary Retention	13 (18.1)			16 (10.3)	2 (0.3)				13 (17)
Acute Kidney Failure					1 (0.2)				
Urinary tract infection	7 (9.7)						3 (5.9)		
Dysuria			2 (2.8)		64 (9.9)			5 (4.8)	

## Data Availability

The data included in this article are available upon request to the corresponding authors.
